# Evaluating and Comparing Behavioural and Electrophysiological Estimates of Neural Health in Cochlear Implant Users

**DOI:** 10.1007/s10162-020-00773-0

**Published:** 2020-11-04

**Authors:** Tim Brochier, François Guérit, John M. Deeks, Charlotte Garcia, Manohar Bance, Robert P. Carlyon

**Affiliations:** 1 Cambridge Hearing Group, MRC Cognition and Brain Sciences Unit, University of Cambridge, 15 Chaucer Road, Cambridge, CB2 7EF UK; 2 Cambridge Hearing Group, Cambridge University Hospitals Foundation Trust, Hills Road, Cambridge, CB2 0QQ UK

**Keywords:** cochlear implants, neural health, neural survival, ECAP, psychophysics, inter-phase gap, polarity effect, multi-pulse integration, computational modelling

## Abstract

Variations in neural health along the cochlea can degrade the spectral and temporal representation of sounds conveyed by cochlear implants (CIs). We evaluated and compared one electrophysiological measure and two behavioural measures that have been proposed as estimates of neural health patterns, in order to explore the extent to which the different measures provide converging and consistent neural health estimates. All measures were obtained from the same 11 users of the Cochlear Corporation CI. The two behavioural measures were multipulse integration (MPI) and the polarity effect (PE), both measured on each of seven electrodes per subject. MPI was measured as the difference between thresholds at 80 pps and 1000 pps, and PE as the difference in thresholds between cathodic- and anodic-centred quadraphasic (QP) 80-pps pulse trains. It has been proposed that good neural health corresponds to a large MPI and to a large negative PE (lower thresholds for cathodic than anodic pulses). The electrophysiological measure was the effect of interphase gap (IPG) on the offset of the ECAP amplitude growth function (AGF), which has been correlated with spiral ganglion neuron density in guinea pigs. This ‘IPG offset’ was obtained on the same subset of electrodes used for the behavioural measures. Despite high test–retest reliability, there were no significant correlations between the neural health estimates for either within-subject comparisons across the electrode array, or between-subject comparisons of the means. A phenomenological model of a population of spiral ganglion neurons was then used to investigate physiological mechanisms that might underlie the different neural health estimates. The combined experimental and modelling results provide evidence that PE, MPI and IPG offset may reflect different characteristics of the electrode-neural interface.

## **INTRODUCTION**

Cochlear implants (CI) restore a sense of hearing to people with sensorineural hearing loss by stimulating the spiral ganglion neurons (SGN) of the auditory nerve. The ability of a CI to transmit speech information partly depends on the condition of the stimulated neural population, which will be referred to as neural health throughout this article. Estimates of neural health patterns along the length of the cochlea may help to guide the optimisation of sound processing strategies, by identifying electrodes in healthy neural regions that might benefit from focused stimulation (Bierer 2010) or electrodes in unhealthy neural regions that could be candidates for deactivation (Garadat et al. 2013; Zhou 2017; Goehring et al. 2019). Several different methods to estimate neural health patterns along the cochlea have been proposed. The present study explores the extent to which three different measures provide converging and consistent estimates of neural health. We consider a combination of both behavioural and electrophysiological measures of neural health.

The behavioural neural health estimates that we evaluated were multipulse integration (MPI) and the polarity effect (PE). MPI is measured either as the slope of the function relating psychophysical detection thresholds to stimulation pulse rate or as the difference between psychophysical detection thresholds at different stimulation pulse rates. As pulse rate is increased, detection thresholds decrease, with the slope being steeper at pulse rates greater than 1000 pps in both animal studies (Kang et al. 2010; Pfingst et al. 2011; Zhou et al. 2015; Pfingst et al. 2017) and studies in CI listeners (Shannon 1985, 1989; McKay and McDermott 1998; Kreft et al. 2004; Zhou et al. 2012, 2015). This characteristic rate-threshold function is consistent with a temporal integration mechanism (Stypulkowski and van den Honert 1984; Shannon 1989; McKay and McDermott 1998; McKay et al. 2003; Carlyon et al. 2005), whereby the neural excitation by consecutive pulses is summed in a sliding integration window. The magnitude of the MPI slope for pulse rates below 1000 pps has been positively correlated with SGN density in guinea pigs (Kang et al. 2010; Pfingst et al. 2011; Zhou et al. 2015). In those studies, guinea pigs were behaviourally trained to complete a detection threshold task, and then split into two groups: one that was implanted after being deafened with neomycin (leading to no residual hearing and low SGN density) and another that was implanted without being deafened (leading to some residual hearing and high SGN density). MPI slopes were significantly larger for the group of guinea pigs that had been implanted without being deafened. For bilateral CI users, between-ear differences in MPI were correlated with between-ear differences in speech perception (Zhou and Pfingst 2014). These results have led to the use of MPI as a behavioural estimate of neural health in humans (Hughes et al. 2014; Zhou and Pfingst 2014, 2016a, 2016b; McKay and Smale 2017; Zhou and Dong 2017; Zhou et al. 2018).

PE is measured as the difference in psychophysical detection thresholds between asymmetric pulse shapes in which the portion where charge that is most concentrated in time is anodic versus where it is cathodic (Carlyon et al. 2013; Macherey et al. 2017). For simplicity, we will describe these charge-balanced asymmetric pulses as ‘anodic’ and ‘cathodic’ pulses, respectively. Modelling studies show that cathodic pulses preferentially stimulate the peripheral processes of an SGN fibre, while anodic pulses preferentially stimulate the central axon (Rattay et al. 2001; Joshi et al. 2017; Resnick et al. 2018; Potrusil et al. 2020). In general, SGN degeneration begins with the peripheral processes and progresses towards the central axon (Spoendlin 1975,Spoendlin 1984; Leake and Hradek 1988; Otte et al. 1978; Hinojosa and Marion 1983; Nadol 1990; Wise et al. 2017). Theoretically, a population of SGN fibres with peripheral degeneration and healthy central axons would have higher threshold for cathodic than for anodic pulses, whereas a population of SGN fibres with healthy peripheral processes and central axons would show a smaller threshold difference between pulse shapes (low or negative PE). At low levels, PE varies across the electrode array within subjects (Macherey et al. 2017; Carlyon et al. 2018; Goehring et al. 2019; Jahn and Arenberg 2019), suggesting that PE at threshold might be a localised indicator of peripheral neural health. Both Jahn and Arenberg (2019) and Mesnildrey et al. (2020) provide psychophysical evidence for PE as an estimate for neural health. Both studies found that PE was correlated with thresholds for symmetric pulses obtained with focused stimulation, which have been shown to be estimates of combined properties of the electrode-neural interface. The *electrode-neural interface* refers to any factor that affects the transmission of information between the implanted electrodes and the neural population, including both neural health and non-neural factors such as electrode position, electrode orientation and impedance. Importantly, this correlation between PE and focused thresholds was significant even when the effects of electrode-modiolar distance (‘EMD’, an estimate of the distance of the electrode from the auditory nerve) were removed. It should also be noted that the model proposed by Rattay et al. (2001) predicts that PE may also be affected by EMD, although we know of no evidence that it correlates with the PE independently of focused thresholds.

We used features calculated from the electrically evoked compound action potential (ECAP) as the electrophysiological estimates of neural health. While many absolute ECAP measures, such as maximum ECAP amplitude (Shepherd and Javel 1997) and slope of the ECAP amplitude growth function (Ramekers et al. 2014; Pfingst et al. 2015), have been positively correlated with neural health in animals, these measures are prone to the influence of non-neural factors such as electrode position (Shepherd et al. 1993) and impedance (Schvartz-Leyzac and Pfingst 2016). Non-neural factors are especially influential in humans, who have larger cochleas and longer durations of deafness compared to other animals, leading to wider electrode-to-electrode variance in electrode position and impedance. To remove the influence of non-neural factors, ratios can be calculated between absolute ECAP features that are measured with different stimulus parameters (e.g. interphase gap (IPG) or phase duration). These differential ECAP measures can potentially partial out the effects of non-neural factors, which are assumed to be the same across stimulus conditions. In separate guinea pig studies, Prado-Guitierrez et al. (2006) and Ramekers et al. (2014) measured the dB difference in current level required to elicit an equal normalised ECAP amplitude at different IPGs, and found that this ‘IPG offset’ was correlated with SGN density. These results have led to measures such as the IPG offset (and similar differential ECAP measures) being commonly used to estimate local neural health along the array in humans (Kim et al. 2010; Schvartz-Leyzac and Pfingst 2016; McKay and Smale 2017; Hughes et al. 2018; Schvartz-Leyzac and Pfingst 2018). We recently evaluated different ways of defining the effect of IPG on neural responses and concluded that the IPG offset provided a robust measure that was minimally affected by non-neural factors (Brochier et al. 2020).

Here, the electrophysiological measure of IPG offset and the behavioural measures of PE and MPI were evaluated and compared in a group of 11 CI users at seven different electrode locations spanning the array. Within-subject and between-subject correlations were evaluated to determine the extent to which the different neural health estimates provide converging information. It was hypothesised that if each of these measures reflects a similar characteristic of neural health, then significant within-subject and between-subject correlations would be found between all of the measures. Further, a computational model of the electrically stimulated auditory nerve (Joshi et al. 2017) was implemented to explore what factors may have contributed to the different neural health estimates.

## **METHODS**

### Participants

Eleven postlingually deafened cochlear implant users completed the series of behavioural and electrophysiological experiments. Permission to conduct the studies was granted by the National Research Ethics committee for the East of England, and subjects provided their written consent to participate. All subjects used devices from Cochlear Limited (Sydney, Australia). Details about each subject’s age, implant type, duration of deafness and aetiology are provided in Table 1.

**Table 1:** Information about the participants in the study

	Age	Implant type	Duration of profound hearing loss before implantation	Duration of implant use	Aetiology
S1	69	CI24RE	48	14	Hereditary
S2	65	CI24RE	13	4	Ear infection, ototoxic antibiotic
S3	66	CI422	18	4	Exposure to loud sounds
S4	57	CI522	17	4	Maternal rubella and ear infection
S5	67	CI522	1.5	3.5	Unknown
S6	78	CI522	5	3.5	Otosclerosis, noise exposure
S7	66	CI512	20	2	Exposure to loud sounds
S8	75	CI24RE	10	15	Scarlet fever, viral infections, and Meniere’s disease
S9	43	CI522	14	2.5	Hereditary
S10	30	Hybrid (residual low frequency hearing)	14	12	Perinatal deafness due to ototoxic antibiotic
S11	57	CI522	3	2	Hereditary

For most subjects, the electrodes tested were, from most basal to most apical, electrodes number 3, 6, 9, 12, 15, 18 and 20. Electrode 3 was disabled for S9 and S10, so electrode 4 was used instead. For each of the tested electrodes, multipulse integration, polarity effect and ECAP amplitude growth functions (AGFs) were measured. Subjects 2, 5 and 6 had no measurable ECAPs. For subjects 2 and 5, PE and MPI data were gathered, and for subject 6, only PE data were gathered.

### Materials

Stimuli for the behavioural measures were generated in MATLAB and delivered to a Cochlear CP910 speech processor using Nucleus Implant Communicator software version 4 (NIC4), provided by Cochlear Limited. Electrophysiological measures were obtained with neural response telemetry (NRT) in Cochlear Custom Sound EP software version 5.2, also provided by Cochlear Limited. All stimuli were checked using a test implant and a digital oscilloscope.

### Multipulse Integration

MPI was measured as the difference in behavioural detection thresholds between 80- and 1000-pulse-per-second (pps) cathodic-leading symmetric biphasic pulse trains for a subset of electrodes across the electrode array. The stimuli were in monopolar (MP) mode with a duration of 0.4 s, a phase duration of 25 μs and an interphase gap of 8 μs.

Before determining thresholds, subjects completed a loudness-scaling procedure for both the 80-pps and 1000-pps stimuli on each electrode. During the loudness-scaling procedure, the level of the stimulus started at 0 CL units and was then incrementally raised in small current steps while the subject gave feedback using a loudness chart (from number 1 for ‘just noticeable’ to number 7 ‘loud but comfortable’). This loudness-scaling procedure was used to ensure that stimulation never exceeded the ‘loud but comfortable’ level reported by the subject.

Thresholds were determined using an adaptive one-up/one-down procedure with no feedback. The participant was instructed to respond when they heard a sound by pressing the spacebar on a computer keyboard. The level of the stimulus started at 90 % of the ‘comfortable’ level reported in the loudness-scaling procedure, and was incrementally lowered or raised, depending on the subject’s response. If the subject responded to a stimulus within 3 s of a stimulus onset, the level was reduced by one step and another stimulus was played. If the subject did not respond to a stimulus within 3 s of the stimulus onset, the level of the stimulus was raised by one step. The procedure stopped when 8 reversals were reached. The initial step size was 4 CL steps (approximately 0.63 dB), and was reduced to 2 CL steps (approximately 0.31 dB) after 2 reversals. The detection threshold for one run was calculated as the mean of the last 6 reversals.

Two runs of detection thresholds were obtained for each electrode and each pulse rate, and the overall detection threshold was calculated as the average of the two runs on each electrode. For each subject, the order of testing the electrodes was randomised for the first run, and repeated in reverse order for the second run. To further minimise effects of testing order on MPI, the first run was obtained for both pulse rates before the second run was obtained, and the order of pulse rates for the second run was reversed. Half the subjects began the test with the 80-pps condition, and half began the test with the 1000-pps condition.

### Polarity Effect

PE was measured as the difference in thresholds between cathodic-centred and anodic-centred quadraphasic (QP-CAAC and QP-ACCA, respectively) pulse trains, at a stimulation rate of 80 pps with a phase duration of 42 μs and an interphase gap of 8 μs. A pulse duration of 42 μs was used rather than 25 μs to ensure audibility, to remain within compliance limits of the device, and to remain consistent with other studies that have measured the polarity effect with QP pulses (Carlyon et al. 2013; Macherey et al. 2017). QP pulses consist of two symmetric biphasic pulses of opposite leading polarity separated by the smallest allowable interphase gap (8 μs). These pulses are similar in principle to triphasic pulses, but comply with the research software for cochlear devices, which only permits symmetric pulse shapes. The loudness-scaling and detection-threshold procedures were identical to those described in the previous section.

### ECAP Amplitude Growth Functions

Symmetric biphasic pulse trains were presented in monopolar mode with a stimulation rate of 80 pps, a phase duration of 25 μs and IPGs of both 8 μs and 40 μs. The recording electrode was always two apical (+ 2) from the stimulating electrode. The forward masking technique was used to remove stimulation artefacts (Brown et al. 1990). The ground electrode was MP1 for the probe and masker pulse and MP2 for the recording electrode. The effective sampling rate was 20 kHz, and the length of the measurement window was 1600 μs. The masking pulse was always presented on the same electrode and at the same current level as the probe pulse, with a masker–probe interval of 400 μs.

Before measuring ECAP AGFs, the loudness-scaling procedure from the previous two sections was completed for each of the tested electrodes. Recording parameters were optimised by measuring a single ECAP at the ‘loud but comfortable’ level for electrodes 3, 12 and 20, with all combinations of recording amplifier gains of 40, 50 and 60 dB, and recording delays of 73, 98 and 122 μs at each electrode. The gain and delay settings that maximised ECAPs were chosen for each subject, and used for all ECAP AGF measurements for that subject. The range over which ECAP AGFs was measured was from level number 2 (‘very soft’) to level number 7 (‘loud but comfortable’), in steps of 3 CL units. A total of 50–100 sweeps were taken at each level for each electrode. N1–P1 peak amplitudes were calculated automatically using Custom Sound EP.

#### Calculating the IPG Offset

**Fig. 1:**
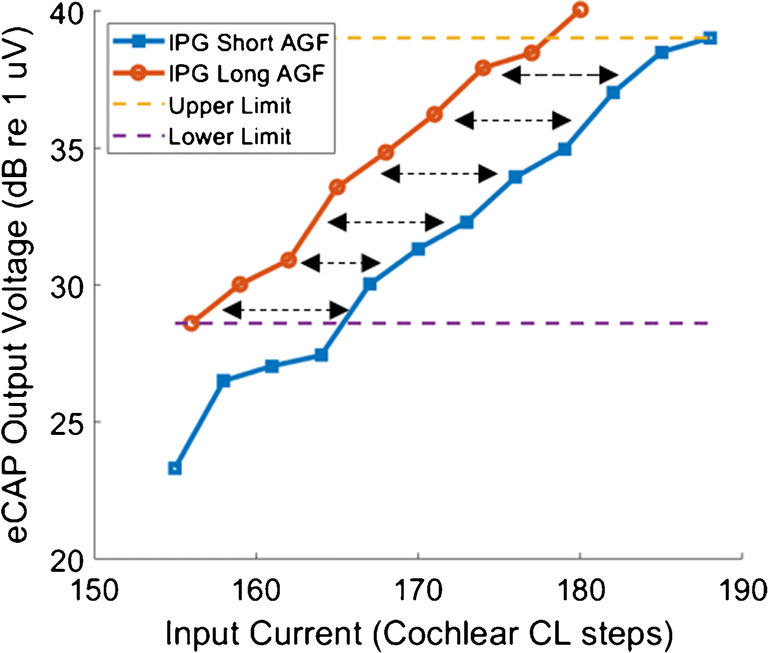
Amplitude growth functions (AGFs) for subject 11, electrode 18, obtained at the 8- and 40-μs IPG (blue and orange lines). The arrows illustrate the ‘IPG offset’ between the two functions, which is defined as the mean difference between overlapping linear portions of the two ECAP AGFs (on a log input–log output scale)

ECAP amplitudes (in dB re 1 μV) were plotted as a function of input current (in dB re 1 μA) for the short IPG of 8 μs and the long IPG of 40 μs. A logarithmic scale was used for both the input and the output because linear coordinates make the difference proportional to the mean, and so differences across electrodes and between subjects are highly susceptible to non-neural factors such as recording electrode and stimulating electrode characteristics (Brochier et al. 2020). The IPG offset was calculated as the average difference (in dB) between the overlapping linear portions of the ECAP AGFs (McKay and Smale 2017). The initial output range over which the IPG offset was calculated was set to the entire overlapping region of ECAP outputs, and was then adjusted to only include the linear portions of amplitude growth by excluding portions of the AGF that were at floor level or saturation level. The method for calculating the IPG offset is shown in Fig. 1. The distance between the ECAP AGFs (indicated by the arrows) was averaged for input currents within the range of the overlapping linear portions of the AGF in steps of 0.1 dB. For input currents where an ECAP measurement was not made, linear interpolation was used between the two nearest measurements. This method was similar to the measurement of the IPG offset used by Prado-Guitierrez et al. (2006), who measured the offset in the regions of 20–80 % of the normalised ECAP AGFs, and by Ramekers et al. (2014), who measured the offset at 50 % of the normalised ECAP AGFs. Both studies showed that their measure of IPG offset correlated with SGN density. Because we were not able to obtain full sigmoidal ECAP AGFs without exceeding the ‘loud but comfortable’ level for the subjects in the present study, the overlapping linear region was used as the closest approximation to the method used in the animal studies.

All between-electrode correlations reported here were calculated using an ANCOVA with one measure as the dependent variable, the other measure as the covariate and the subject as a random factor (Bland and Altman 1986). This is mathematically equivalent to subtracting, for each measure and electrode, the mean value for that measure obtained for that subject across all electrodes, and then performing a Pearson correlation. This latter method is used when plotting correlations, so that each axis shows a normalised value relative to the mean for each subject.

## **RESULTS**

**Fig. 2:**
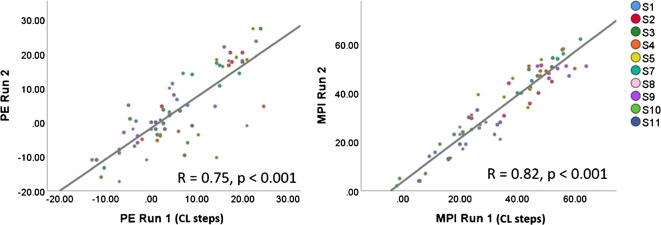
Test–retest reliability was tested by measuring the correlation between the first and second run of the PE measurement (left panel) and MPI measurement (right panel). The Pearson correlation coefficients and *p* values were calculated from an ANCOVA with subject as a random factor. Each axis shows the value for each subject and electrode relative to the mean value across electrodes for that run number

Run 1 and run 2 were highly correlated for both PE (*R*(10) = 0.75, *p* < 0.001) and MPI (*R*(9) = 0.82, *p* < 0.001), suggesting good test–retest reliability for the behavioural measures (Fig. 2). Test–retest reliability was not explicitly measured for the IPG offset measure, but 50–100 sweeps were averaged to obtain each ECAP AGF from which the IPG offset was calculated.

An analysis of covariance (ANCOVA) with subject as a random factor was used to evaluate the correlations between PE, MPI and the IPG effect. No significant correlations were found between any of the proposed neural health estimates. PE was not correlated with MPI (*R*(9) = 0.11, *p* = 0.385), MPI was not correlated with the IPG effect (*R*(7) = − 0.29, *p* = 0.058) and PE was not correlated with the IPG effect (*R*(7) = 0.02, *p* = 0.887). The absence of correlations shows that the within-subject pattern of one neural health estimate across the electrode array is not predictive of the within-subject pattern of another neural health estimate across the electrode array, at least in our subject population.

**Fig. 3:**
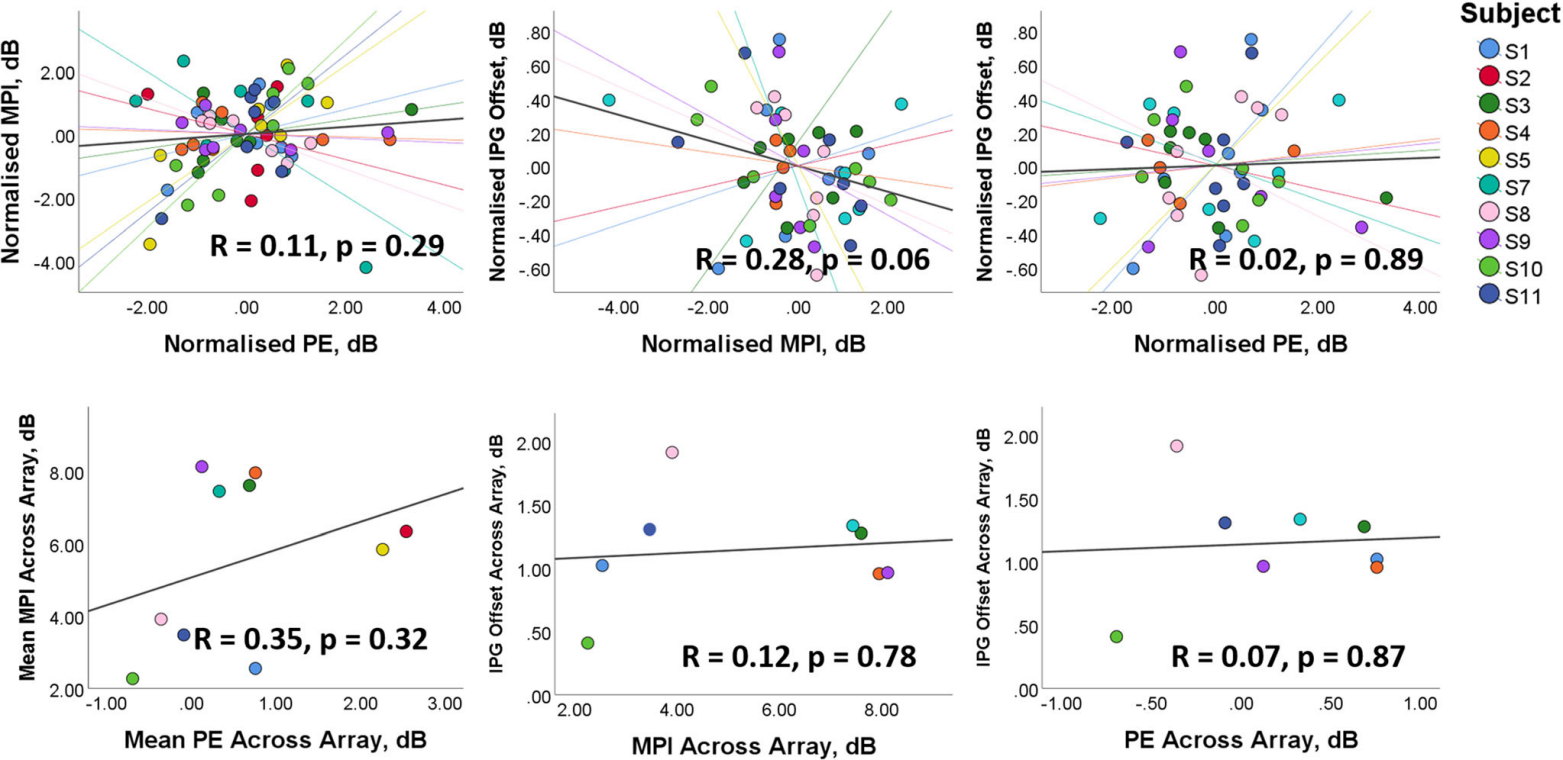
The figures show the correlation analyses between the different neural health metrics. The upper panels show within-subject correlations across the electrode array, and the lower panels show the between-subject correlations of the means across the electrode array. For the between-electrode correlations, each axis shows the value of for each subject and electrode relative to the mean value across electrodes for that measure. The lines are least-squares regression lines for each subject’s data normalised to their mean across electrodes. None of the neural health metrics were significantly correlated, either for within-subject or between-subject comparisons

Figure 3 shows the correlations between MPI and PE, MPI and IPG effect, and PE and IPG effect. In the upper panels, the mean value across electrodes for each subject was subtracted from the measurement at each electrode. The lower panels show the correlations between the mean values across electrodes for each subject. A Fisher *R* to *z* transform was used on the correlation coefficients for each individual to calculate the 95 % CI for the range of across-electrode correlations for each comparison of neural health estimates. The 95 % CI for the range of correlation coefficients for MPI and PE was 0.03 ± 0.4; for MPI and IPG effect, it was − 0.27 ± 0.33; and for PE and IPG effect, it was 0.04 ± 0.39. All of these ranges encompass zero, reflecting the lack of statistical significance of the correlations.

The between-subject correlations of the mean of each neural health estimate across the array were also calculated and are plotted in the bottom row of Fig. 3. No significant correlations were found between any of the estimates of neural health. Mean MPI across the array was not correlated with mean PE (*R*(9) = 0.35, *p* = 0.322) or mean IPG effect (*R*(7) = 0.12, *p* = 0.783), and mean PE was not correlated with mean IPG effect (*R*(7) = 0.068, *p* = 0.873).

**Fig. 4:**
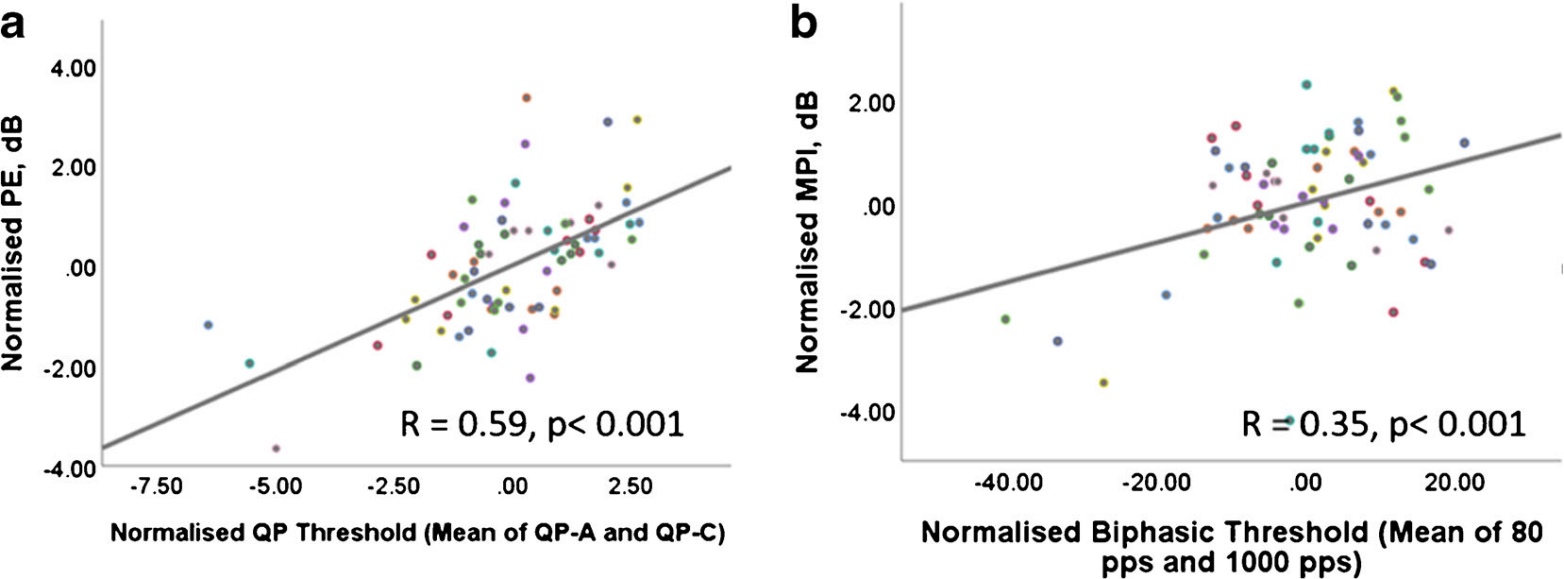
Pearson correlation analysis between PE and QP thresholds (left panel), and MPI and biphasic thresholds (right panel). PE was positively correlated with the mean QP threshold and MPI was positively correlated with the mean biphasic threshold

As noted in the ‘Introduction’ section, a number of previous studies have shown that the PE is positively correlated with the average threshold obtained with both polarities (Carlyon et al. 2018; Jahn and Arenberg 2019) or with thresholds obtained with symmetric pulses (Mesnildrey et al. 2020). Our results replicate both of those findings: PE was positively correlated with the mean of QP-CAAC and QP-ACCA thresholds (*R* = 0.59, *p* < 0.001, Fig. 4a), and with 80-pps thresholds obtained with symmetric biphasic pulses (*R* = 0.40, *p* < 0.001). MPI was correlated with the mean of the 80-pps and 1000-pps biphasic thresholds (*R* = 0.35, *p* < 0.001, Fig. 4b).

### Computational Modelling of the Neural Health Estimates

A phenomenological model of a spiral ganglion neuron (Joshi et al. 2017) was used to investigate the physiological mechanisms that might underlie the different neural health estimates. The model from Joshi et al. (2017) was well suited for this investigation for several reasons. It models each neuron using both a peripheral process (the entire pre-somatic region of the SGN) and a central axon (the entire post-somatic region of the SGN). The peripheral process and central axon are characterised by separate exponential integrate-and-fire point-processes, allowing us to simulate neural degeneration by either disabling the peripheral processes entirely, or by increasing the membrane capacitance and reducing the membrane conductance to model demyelination/degeneration of the peripheral process and/or central axon (Resnick et al. 2018). The model also includes sub-threshold and supra-threshold feedback loops, which account for temporal properties of neurons (Boulet et al. 2016), allowing the investigation of various pulse shapes (e.g. different IPGs and QP pulses) and stimulation rates (e.g. 80 pps and 1000 pps). Finally, the use of this phenomenological model was preferable compared to other biophysical models (Colombo and Parkins 1987; Rattay et al. 2001; Briaire and Frijns 2005; Smit et al. 2010), which have been shown to inaccurately estimate the effect of pulse rate on detection thresholds (Bachmaier et al. 2019). Because of the model’s computational efficiency, we were able to generate populations of 500–1000 neurons with various means and SDs of firing thresholds. For each of the neural health estimates, we tested the effect of number of neurons, mean firing threshold, SD of the firing thresholds, presence of the peripheral processes and central axon demyelination. A more detailed description of the model is provided by Joshi et al. (2017). The MATLAB (version R2019b) source code can be found at https://github.com/neurongeek/ANF-model. It has been shared with the permission of the author, Dr. Suyash Joshi.

#### IPG Offset

In the computational model, the IPG offset was unaffected by the number of neurons, or by the mean and SD of the firing thresholds of the neural population. It was very subtly affected by removing the peripheral processes. The greatest reduction in IPG effect occurred by disabling the peripheral system and modelling demyelination of the central axon by increasing the membrane capacitance and reducing the membrane conductance.

**Fig. 5:**
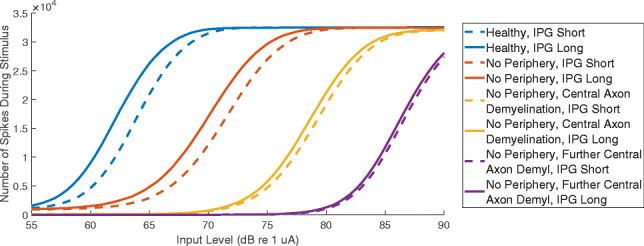
Modelling results for the IPG offset. The number of spikes generated by a population of 1000 neurons is shown as a function of input current level. The stimulus was identical to the electrophysiological experiment, with a duration of 0.4 s, a stimulation rate of 80 pps, and an IPG of either 8 μs (dotted lines) or 40 μs (solid lines). The different colours represent different amounts of SGN degeneration

In Fig. 5, the number of spikes generated by a population of 1000 neurons is shown as a function of input current level. The stimuli were identical to those used for the IPG offset measure in the behavioural portion of the experiment (duration of 0.4 s, 80 pps stimulation rate, 25 μs phase duration, IPGs of 8 and 40 μs). The 8-μs and 40-μs IPGs are shown by dashed and solid lines, respectively. The IPG offset is largest for the healthy neural population (blue lines) and is only slightly reduced by removing the peripheral processes removed (orange lines). When peripheral processes are removed and central axon demyelination is applied, the IPG offset decreases, as shown by the yellow and purple lines. For the yellow lines, central axon demyelination was modelled by doubling the membrane capacitance and halving the membrane conductance, and for the purple lines, membrane capacitance was quadrupled and conductance was quartered. These values were chosen to qualitatively assess the interaction between the IPG effect and neural degeneration, and so the absolute value of the shift in amplitude growth functions may be exaggerated compared to that of real CI listeners.

**Fig. 6:**
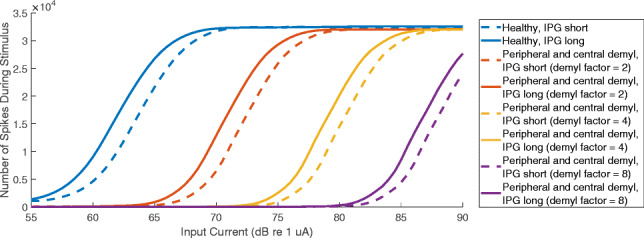
Modelling results for the IPG offset, with simultaneous rather than retrograde neural degradation. The number of spikes generated by a population of 1000 neurons is shown as a function of input current level. The stimulus was identical to the electrophysiological experiment, with a duration of 0.4 s, a stimulation rate of 80 pps, and an IPG of either 8 μs (dotted lines) or 40 μs (solid lines). The different colours represent different amounts of SGN degeneration

It is important to note that, in some cases, degeneration of the peripheral processes and central axon occur simultaneously, rather than sequentially (Wise et al. 2017; Ramekers et al. 2020). Figure 6 shows a separate IPG offset modelling analysis where the peripheral process and the central axon were simultaneously demyelinated by multiplying the membrane capacitance and dividing the membrane conductance by ‘demyelination factors’ of 2 (orange lines), 4 (yellow lines) and 8 (purple lines). This differed from the previous model because the peripheral process remained enabled for all simulations. While the IPG offset was still reduced with increasing demyelination, the effect was less pronounced than for the case where central axon demyelination was applied after the peripheral processes were disabled.

These modelling results suggest that IPG offset might be a measure of central axon demyelination, particularly when the peripheral processes are completely degenerated. In other words, the IPG offset becomes most sensitive when the amount of neural degeneration is relatively severe. The main support in the literature for the use of IPG offset as an estimate for neural health are the studies by Prado-Guitierrez et al. (2006) and Ramekers et al. (2014), which both showed correlations between SGN density and IPG offset. It is likely that SGN density, as estimated in those studies, covaries with central axon demyelination. SGN density was estimated by counting the nuclei of SGN fibres, which lie in the soma between the peripheral processes and the central axon. Whether we assume retrograde or simultaneous SGN degeneration, the amount of central axon demyelination is probably related to the number of degenerated soma and, consequently, the calculated SGN density. Interestingly, the model suggests that the IPG offset is not directly affected by the *number* of remaining neurons, but rather by the change in the *properties* of those remaining neurons.

#### Polarity Effect

**Fig. 7:**
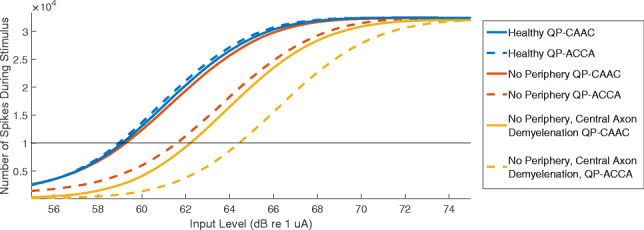
Modelling results for the polarity effect. The number of spikes generated by a population of 1000 neurons is shown as a function of input current level. The stimulus was identical to the behavioural PE experiment, with a duration of 0.4 s, a stimulation rate of 80 pps, and cathodic-centred (dotted line) or anodic-centred (solid line) quadraphasic pulses. The different colours represent different amounts of SGN degeneration

Like the IPG offset, the computational model of PE was unaffected by the number of neurons, or by the mean and SD of the firing thresholds of the neural population. PE could however be modelled by disabling the peripheral processes, or modelling degeneration of the peripheral processes by increasing membrane capacitance and reducing membrane conductance. Figure 7 shows the number of spikes generated by a modelled population of 1000 SGN fibres as a function of input current, throughout the 0.4-s duration of cathodic-centred (dotted lines) and anodic-centred (solid lines) QP stimuli (stimulation rate 80 pps, phase duration 42 μs, IPG 8 μs). It was assumed that the detection threshold was reached when a certain number of spikes were generated. For this example, the threshold number of spikes was set at 10,000, but any choice of number would lead to a qualitatively similar result. For the neural population with healthy peripheral processes, the threshold for cathodic-centred pulses was slightly lower than the threshold for anodic-centred pulses. For the population without peripheral processes, the threshold for anodic-centred pulses was approximately 2 dB lower than the threshold for cathodic-centred pulses. When (additionally) the central axon was demyelinated, PE was unaffected.

This result suggests that PE may be a measure of the survival of the peripheral processes, consistent with other modelling studies (Rattay et al. 2001; Resnick et al. 2018; Potrusil et al. 2020). The modelling implies that unlike IPG offset, PE is *not* further affected by central axon demyelination, which could help to explain why IPG offset and PE were not found to be correlated.

#### Multipulse Integration

**Fig. 8:**
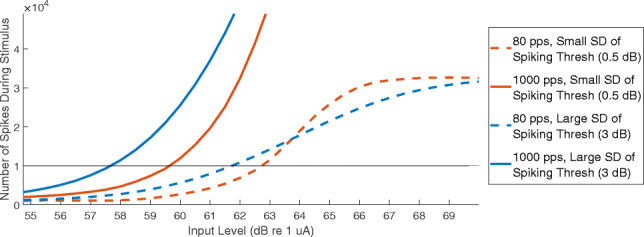
Modelling results for multipulse integration. The number of spikes generated by a population of 1000 neurons is shown as a function of input current level. The stimulus was identical to the behavioural experiment, with a duration of 0.4 s and a stimulation rate of 80 pps (dotted line) or 1000 pps (solid line). The different colours represent different SDs of firing thresholds for the modelled neural populations, with orange corresponding to a small SD of 0.5 dB and blue corresponding to a large SD of 3 dB

The computational model of MPI was affected by the SD of the firing thresholds of the population of neurons, with larger MPIs associated with larger SDs. Figure 8 shows the number of spikes as a function of input current level for pulse trains with stimulation rates of 80 pps (dotted lines) and 1000 pps (solid lines). The blue curves were measured for a population of 1000 SGNs with a 3 dB SD of firing thresholds, and the orange curves were measured with a population of 1000 SGNs with a 0.5 dB SD of firing thresholds. Similar to the modelling of PE, the detection threshold was assumed to occur at 10,000 neural spikes, although the results would be qualitatively similar for any number of spikes. The difference between thresholds for 80 pps and 1000 pps pulse trains was approximately 4 dB for the population with a large SD of firing thresholds, and 3 dB for the population with a small SD.

**Fig. 9:**
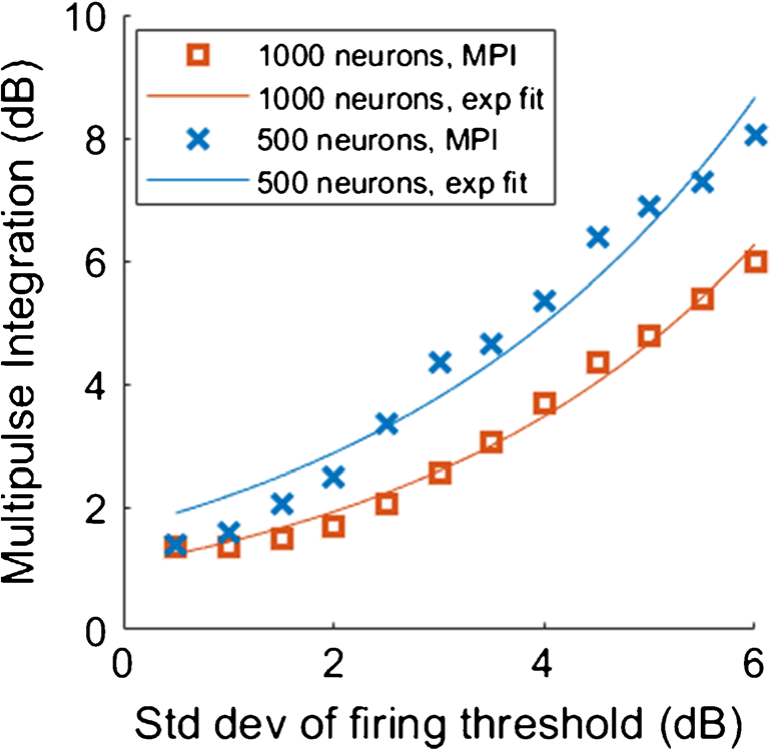
Multipulse integration as a function of the SD of firing thresholds, for populations of 1000 neurons (orange line) and 500 neurons (blue line)

MPI was also affected by the number of neurons and by the removal of the peripheral processes. However, the direction of these effects was unexpected, with higher MPI for lower number of neurons and for the population without peripheral processes. When populations of 500 and 1000 neurons were tested (with equal SDs), the smaller population of 500 neurons had a larger MPI than the larger population of 1000 neurons (3.8 dB vs. 2.9 dB). When the peripheral processes were removed, MPI increased for both the 500- and 1000-neuron populations (to 5 dB and 4.6 dB, respectively). Figure 9 shows MPI as a function of the SD of firing thresholds for populations of 1000 neurons and 500 neurons.

## **DISCUSSION**

MPI, PE and the IPG offset, all proposed measures of neural health, were evaluated and compared in 11 CI users. There were no significant correlations between the neural health estimates for either within-subject comparisons across the electrode array or between-subject comparisons of the means. The results therefore suggest that these neural health estimates do not reflect the same characteristic of neural health.

One clear contributor to the lack of within-subject correlations between the IPG offset and our behavioural measures is the different range of levels used to make these measurements. The IPG offset is measured well above threshold, at levels that are high enough to elicit ECAPs. In contrast, MPI and PE were measured at psychophysical detection thresholds. Therefore, the spread of current was likely larger and the stimulated neural population would have been broader for the IPG offset measurement than the behavioural measurements. However, the different overall levels used to measure IPG offset and our behavioural measures does not fully explain why we failed to see a between-subject correlation between those measures. Even if the stimulated neural population were broader for the IPG offset measurement, one would expect that the average IPG offset would correlate with the average behavioural measures between subjects, if they all reflected the same characteristics of neural health.

An explanation for the lack of between-subject correlations between the neural health measures, supported by both our experimental and modelling data, is that the different neural health measures reflect different characteristics of the neural population. A computational model was used to explore what factors may have contributed to the behavioural and electrophysiological neural health estimates in this experiment, and to identify distinct mechanisms that may have affected one measure independently of the other measures. Computational modelling provides a unique framework whereby different neural health factors can be assessed independently, which cannot be done easily in animal studies, or at all in human experiments. For example, the absolute number of neurons could be adjusted while maintaining all other neural properties, or the peripheral processes could be degenerated independently of the central axon. Our modelling results suggest that IPG offset may be related to central axon demyelination (particularly when the peripheral processes were disabled), PE may be related to health of the peripheral processes and MPI may be related to the SD of fibre thresholds.

At first, it may seem surprising that measures such as IPG offset and MPI were not affected by absolute neuron count in the computational model, because those measures have been shown to be correlated with SGN density in animals. However, it is has been shown that the number of nerve fibres (SGN density) covaries with neural degeneration/demyelination in animal studies. The modelling results suggest that the properties of the nerve fibres, as opposed to the absolute number of nerve fibres, influence the neural health measures. These results do not necessarily conflict with the animal studies that have shown a correlation between SGN density and IPG offset or MPI, and in fact, it may serve to expand on those data by probing the anatomical cause of the effect in more detail.

The idea that the IPG offset might reflect neural properties rather than the total number of neurons is supported by a recent study that measured the ECAP IPG offset in children diagnosed with cochlear nerve deficiency (CND) (Skidmore et al. 2020). CND is defined as a small or absent cochlear nerve, and CI outcomes for this pathology are generally poor. However, Skidmore et al. (2020) showed that the IPG offset was significantly larger for the CND group compared to the group of CI users without CND, indicating that the degree of neural degeneration was less in the group with CND than the non-CND group. This result is consistent with temporal bone studies, which have shown that the remaining neurons in CND patients (while sparse) are not necessarily degenerated (Ylikoski and Savolainen 1984). In another study, a cochlear microphonic was measured in 9 out of 13 ears with CND (Buchman et al. 2006), indicating the presence of innervated inner and outer hair cells. Hence, compared to typical CI users with severe sensorineural hearing loss, the CND group might have fewer but healthier neurons. The properties of those neurons, and not the number, are reflected by the significantly larger IPG effect in the CND group compared to the non-CND group.

‘Neural health’ is a broad term, composed of many potentially independent factors including SGN density, the distribution of firing thresholds, demyelination and degeneration of peripheral processes and/or the central axon, and temporal properties of the remaining SGNs. In animal studies, many of the factors have been shown to covary, but in humans, there is far more between-subject and within-subject variance in terms of aetiology and electrode-neural interface, which may interfere with the typical patterns of covariance that we observe in animals. For example, the computational model of MPI showed that it was affected by the SD of firing thresholds of the neural population. While the SD of firing thresholds would certainly be affected by the health of the neural population, it could also be affected by current spread and spatial selectivity.

MPI has been shown to be related to spatial selectivity, with higher MPI in monopolar mode compared to bipolar mode (Zhou et al. 2018), and higher MPI on electrodes that produce a wider spatial spread of excitation compared to those that produce a narrow spread (Zhou and Pfingst 2016b). In addition, in our behavioural results, MPI was positively correlated with the mean detection threshold for 80-pps and 1000-pps biphasic pulse trains, suggesting that higher current levels (higher current spread) lead to higher MPI. If the spread of excitation at a particular electrode were relatively wide compared to other electrodes, then the distribution of membrane voltages at nearby SGN fibres would also be wider, leading to a larger SD of firing thresholds. One contributing factor to the spread of excitation is the EMD. In guinea pigs, the EMD remains relatively constant across the electrode array, and variation in MPI between groups of animals deafened for different amounts of time is largely influenced by the health of the SGN fibres. In humans, EMD varies considerably along the length of the cochlea (Long et al. 2014), and the number of recruited nerve fibres (and the SD of their firing thresholds) would depend not only on the health of the nearby SGN fibres but also on the spread of excitation. However, a recent study by Schvartz-Leyzac et al. (2020) measured EMD and MPI at every electrode along the array in 11 CI users, and found that MPI was not correlated with EMD. While other factors, such as electrode impedance or fibrous tissue growth around the electrode, could still contribute to spatial selectivity and hence MPI (MPI in our study was correlated with impedance (*R* = 0.31, *p* = 0.015)), the result by Schvartz-Leyzac et al. (2020) indicates that MPI in most cases might be dominated by characteristics of the neural population.

In that same Schvartz-Leyzac et al. (2020) study, the IPG effect on ECAP AGF slope was measured for the same electrodes for which MPI was measured. The IPG effect on ECAP AGF slope differs from the IPG offset, in that its magnitude depends on absolute ECAP amplitude, and is therefore affected by both neural and non-neural factors (Brochier et al. 2020). That being said, the neural factors contributing to the IPG effect on ECAP AGF slope presumably involve similar underlying mechanisms to the IPG offset. Consistent with our experimental results, Schvartz-Leyzac et al. (2020) found no within-subject correlations between MPI and IPG effect on ECAPs. They draw similar conclusions to the present study, attributing the lack of correlation to differing underlying neural mechanisms between the effect of IPG on ECAP slope and MPI. Considering our computational model, along with the results of Schvartz-Leyzac et al. (2020), MPI is more related to variability of neural thresholds in the neural population, while the effect of IPG is reflective of the central axon characteristics, which might affect the response of the neuron to a single biphasic pulse.

The modelling results also suggest that the IPG offset is related to overall neural excitability. As IPG offset decreases, neural excitation decreases, as observed by the shifting of the AGFs towards the right as the IPG offset reduces. Physiologically, the interaction between IPG offset and neural excitability makes sense. If the IPG offset is related to biophysical properties of the central axon, neurons with a larger IPG offset would probably also be more responsive to electrical stimulation. In humans of course, we cannot measure neural excitability directly; absolute measures such as maximum ECAP amplitude, ECAP threshold or ECAP AGF slope are impacted by both neural and non-neural factors. In animal studies, where non-neural factors such as impedance and EMD are less influential, the aforementioned absolute measures are predominantly influenced by neural excitability. In those data, we see some evidence that the IPG offset is related to neural excitability. The Prado-Guitierrez et al. (2006) data, for example, showed a relationship between the IPG offset and the mean 50 % point on the AGFs for IPGs of 8 and 58 μs (on a logarithmic input and output scale), although the correlation did not reach significance (*R* = − 0.64, *p* = 0.061, *N* = 9).

An alternative hypothesis is that not all of these measures reflect neural health. While multiple animal studies have linked neural health to MPI (Kang et al. 2010; Pfingst et al. 2011; Zhou et al. 2015; Pfingst et al. 2017) and IPG offset (Prado-Guitierrez et al. 2006; Ramekers et al. 2014), the ability of PE to predict neural health has only been demonstrated in modelling studies (Rattay et al. 2001; Joshi et al. 2017; Resnick et al. 2018; Potrusil et al. 2020) and postulated to underlie results in human CI listeners (Macherey et al. 2017; Carlyon et al. 2018; Goehring et al. 2019; Jahn and Arenberg 2019; Mesnildrey et al. 2020).

Animal studies that have investigated PE have not shown the expected relationship between PE and duration of deafness (Macherey and Cazals 2016) or location of micro-lesions (Konerding et al. 2020). Macherey and Cazals (2016) measured the PE in deafened guinea pigs through inferior-colliculus recordings, and found no significant effect of duration of deafness (from 1 week up to 1 year) on PE. Konerding et al. (2020) applied micro-lesions to either the peripheral processes, the soma or the central axon in guinea pigs, and measured PE. Surprisingly, a negative PE (which has been proposed to indicate *better* peripheral neural health) was found in the group of guinea pigs with lesions in the soma or the cell body, compared to guinea pigs without lesions. It should be noted that the present study used biphasic pulses, which have not shown significant effects of polarity in humans. In addition, in the process of applying the lesions, Konerding et al. (2020) may have opened up a current pathway for both the cathodic and anodic-leading biphasic pulses to stimulate the central axon directly, which would negate any PE related to cathodic stimulation of the peripheral processes. The lack of histological evidence for PE as a measurement of peripheral health is likely due to morphological differences between humans and guinea pigs, in both the cochlea and in the neurons themselves. The smaller cochlea of the guinea pig may lead to central axon activation for both cathodic and anodic pulses, which would reduce or completely abolish the PE. At the neural level, guinea pigs have myelinated soma, compared to the unmyelinated soma of the human, which again would cause differences in the site of activation (Rattay et al. 2001). The above differences make the PE inherently difficult to validate in animal studies, compared to IPG offset or MPI. That being said, it has been repeatedly shown in human studies that PE is heterogeneous along the electrode array, and PE was found to be significantly different in the pre-lesion and post-lesion guinea pigs in the Konerding et al. (2020) study. Therefore, there must be some factor that affects PE differently along the cochlea, and there is some evidence that this factor is neural.

### Limitations of the Modelling

It should be emphasised that the model was used as an exploratory method to better understand factors that contributed to the different neural health estimates. The model was based on data from feline SGNs (Miller et al. 1999; Shepherd and Javel 1999; Miller et al. 2001), so the exact values of the results may differ from studies in human CI listeners. In addition, the parameters used to model peripheral degeneration and central axon demyelination were coarse approximations of the real biophysical processes. Nevertheless, the model does help to explain how the different neural health estimates could vary independently of one another.

Another limitation of the model was that it did not evaluate the contribution of inner hair cells (IHCs). In animal studies, MPI has been shown to be larger in animals with surviving IHCs (Zhou et al. 2015; Pfingst et al. 2017), and ECAPs have been shown to have lower maximum amplitudes and shallower AGF slopes in the presence of IHCs (Hu et al. 2003). The reason that IHC survival was not included in the model was because a computational model for electrically stimulated IHCs is not publicly available, to our knowledge. It was assumed that most of our CI users had minimal IHC survival (Hinojosa and Marion 1983), with the exception of S10, who has a hybrid implant and residual low-frequency hearing. Interestingly, S10 showed a large jump in MPI for electrodes 12–20, the electrodes closest to the apex of the cochlea where the surviving IHCs are most likely to be present.

#### Relation to Estimated Duration of Profound HL before Implantation

A significant correlation was found between mean MPI across the electrode array and duration of deafness (*R* = − 0.725, *p* = 0.018). No significant correlations were found between duration of deafness and PE (*R* = − 0.401, *p* = 0.221) or IPG offset (*R* = 0.280, *p* = 0.405). It is important to note that the duration of profound hearing loss before implantation is difficult to measure precisely and relies on self-report from the participants. Especially in cases of slow, progressive hearing loss, it is difficult to identify the exact time point at which profound hearing loss began.

#### Future Considerations for Neural Health Estimates

The personal customisation of CI speech processing based on individual neural characteristics is important for optimising outcomes in CI users. As with other precision medicine, the benefits of a treatment rely upon the accuracy of the diagnostics. Neural health estimates have become increasingly popular, but a consensus has not been reached on the best estimates to use. The present study suggests that different neural health estimates are not interchangeable, as they might measure different properties of the electrode-neural interface. In some sense, this distinctiveness could be advantageous because each of the measurements provides unique information about neural health. For example, in each of our tested subjects, IPG offset and PE could be used in combination to estimate the degree of peripheral and central SGN degeneration along the length of the array. However, due to resource limitations in a clinical setting, it would be more desirable to have a single objective measure that could quickly scan the array and determine the quality of the electrode-neural interface.

Important new information may come from past and future attempts to use neural health estimates to optimise speech processing strategies. While some studies have shown improved speech-in-noise perception by deactivating electrodes with poor modulation detection (Garadat et al. 2013) or high low-rate thresholds (Zhou 2017), a similar strategy that deactivated electrodes with a large PE did not provide any benefit (Goehring et al. 2019). To our knowledge, there has not been an electrode deactivation strategy that has used the IPG offset. Focused stimulation strategies (Arenberg et al. 2018; de Jong et al. 2019) might also benefit by selecting which electrodes to focus and which electrodes to disable based on some neural health estimate.

## **CONCLUSION**

The combined experimental and modelling results provide evidence that PE, MPI and IPG offset reflect different characteristics of the electrode-neural interface. No correlations were found between the different neural health estimates, for either within-subject comparisons across the electrode array, or for between-subject comparison of the means. Modelling results suggest that PE may be related to peripheral neural health, IPG offset may be related to central axon demyelination and MPI may be related to the SD of firing thresholds (which in turn may be affected by spread of excitation).

## Data Availability

A complete table of all results for all subjects can be found at https://github.com/tjbrochier/Neural-Health-Measures-2020. The MATLAB (version R2019b) source code can be found at https://github.com/neurongeek/ANF-model. It has been shared with the permission of the author, Dr. Suyash Joshi.
